# INCLUSIVE CROSS-CULTURAL LEARNING: END-OF-LIFE CRITICAL CARE ACROSS KOREAN AND AMERICAN LITERATURE

**DOI:** 10.1093/geroni/igae098.2390

**Published:** 2024-12-31

**Authors:** Soo Hyun Kim, Rebecca Wright

**Affiliations:** Johns Hopkins University, Baltimore, Maryland, United States; Johns Hopkins University, Baltimore, Maryland, United States

## Abstract

**Background:**

Korean Americans are the fastest growing racial-ethnic population in the US, however, demographic reporting often groups by race, prohibiting exploration of ethnic and cultural needs. This represents a challenge for clinicians tailoring end-of-life care in critical care environments.

**Objectives:**

Report the state of the science from a scoping review of Korean and American end-of-life critical care literature.

**Methods:**

A comprehensive scoping review of the literature was performed in 2024 following the Joanna Briggs methodology, across seven databases: PubMed, CINAHL, EMBASE, Web of Science, KCI, Global Index Medicus, and KoreanMed. Articles were included in English and Korean with no time limit.

**Results:**

Exploration of end-of-life critical care was limited: all studies were descriptive. Korean articles (N=23) highlighted minimal advanced care planning, physician-led care, and an absence of direct patient perspectives. US (N=26) literature revealed a gap in critical care content but included older adult’s hypothetical perspectives of palliative care. Differences were noted in preferences for end-of-life interventions between Korean Americans and other racial-ethnic groups. Some contradictions were noted in Korean American perspectives where life-sustaining treatments were viewed positively but not wanted, yet with an expectation that all treatment options should be pursued out of filial piety.

**Conclusion:**

There is substantial need for research into end-of-life care needs in critical care environments to improve inclusion and equity for Korean Americans. Korean literature provides some insights into cultural norms the US could draw from. However, Korean Americans may have different needs to Koreans, requiring further exploration and intervention development.

